# Palmistry Notes

**Published:** 1887-09-24

**Authors:** 


### Amusements for Convalescents.

### PALMISTRY NOTES.

(*Continued from p. 397*).

Hands are divided into two *classes*, apart from the seven *types*, which will be treated of later on—those with smooth fingers, which when spread out show no development of the joints, and those with knotty fingers, which when parted show the outline of the joints distinctly.

*Smooth-fingered* people are guided by instinct, they are more poetical and less practical than those with knotted fingers; and if the hand be soft and the fingers pointed, there will be an inherent tendency to indolence, and a habit of taking up things by fits and starts, which must be guarded against. *Smooth soft hands*, with *spatulate* spread-out tips, indicate a special weakness in this direction.

This *spatulation* of the finger-tips always shows activity, but in a hand of the order we are considering it shows a constant *desire* for movement, which culminates in no particular good, unless there is a long first joint—*will*—to the thumb, when there will be power to combat this want of sustained effort. If, however, the palm be much rayed, and the thumb small and weak, the indication is conclusive. Smooth-fingered, soft-handed people are also nervous and excitable; they delight in the marvellous and the mystic. With a *very* soft hand and a much rayed palm there will be hysterical and hypochondriacal tendencies.

People whose fingers are knotted have more reason and reflection in their character than smooth-fingered subjects. With the *first joint prominent* we get *mental order*, with the second *material*, while with *the development of both joints* we find reason, calculation, deliberation, reflection, punctuality, and order. With a high mount of the moon found at the side of the hand, extending from the wrist to within an inch of the base of the little finger, we have a love for the sublime and beautiful in music and poetry. With the *upper joint* only developed, *reason will predominate* everything in a character; if very prominent there is always much talent, but the lines in the palm must be firm and clear and the *thumb strong*, otherwise there will be an absence of heart and feeling which will render the subject prone to see things in their rational and material aspect only.

It is unnecessary to say to which of the two classes of mankind the smooth and the knotted hands belong. Dreamers and workers abound on all sides, and we can do without neither; but to the knotted hands is given the power to develop and carry out the inspirations of their more *spiritual* but less persevering brethren. It must not be overlooked that though the chief characteristic of a hard and knotted hand is *activity*, mental and physical, and the leading feature in the character of a *smooth-fingered, soft-handed* subject is their tendency to *indolence*, still, by prolonged illness and consequent inaction, a hard, knotted, active hand may assume temporarily the *consistency* of one that is naturally smooth and soft. This is a fact in favour of the theory that the soft texture of a hand is indicative of *physical* laziness, for by its *enforced* idleness, a hard, strong hand becomes weak and soft; for in proportion as the body is at rest does the mind become active, and so for the nonce the hard-handed practical subject *assumes* a characteristic which is *natural* to the mentally active but physically indolent man or woman. With returning strength the hand regains its former strong texture, and the subject resumes those active occupations which from the nature of the hand he is so specially inclined to and fitted for.

*Large hands* show an appreciation of detail, power of analysis, with a capacity for the performance of fine and delicate work. And it may be consolatory to those people to whom the possession of a large hand has not been a source of unmixed delight, to know that, as the Greeks had no sense of beauty without utility, a large hand was held by them in high estimation.

*Small hands* indicate an admiration for large and colossal works, and a talent for synthesis; while to the medium-sized hand is given the power of entering into subjects in detail, and of grasping it fully as a whole.

In addition to the study of the outline of the fingers generally, there are also certain indications specially attached to the formation of each finger in particular, which before dismissing this branch of the subject it may be interesting to touch briefly upon.

*Long first finger* with pointed tip indicates intuition, pride, strong religious tendencies; if longer than the second finger, ambition will be the ruling impulse; and if the hand is good in other developments, ambition for the highest good will predominate. Such subjects by their intuitive and reflective powers will seek for perfection in all things they may undertake.

*Very long first finger* in a bad hand is indicative of overwhelming pride, which will degenerate into vanity and ostentation, while ambition becomes mere self-seeking.

In a soft hand with smooth fingers a spatulation of a very long first finger gives false judgment, with a delight in the marvellous and uncanny.

*Short first finger*, activity, abruptness, spontaneity.

*Second finger long and flat*, we find melancholy brooding, love of solitude, and if *twisted*, revenge and moroseness; *square*, gravity and dignity. The *spatulate* is the best termination for the second finger, for *then* we shall find activity, which, tempered by reflective power, will result in good and useful action. This finger is very rarely *pointed*, but when it is, the impulse and intuition shown by this formation will mitigate the morbid tendencies indicated above.

*Third finger should be long* to have good significations; we then find love of and ambition in art, boldness, enterprise, research; if as long as the second or middle finger, artistic instinct and talent will prevail over all impediments, and lead to a successful issue in any path chosen. *Shortness* of this finger gives a material turn to the instincts of art. With a long third phalange to this finger love of wealth will be present.

*Fourth finger short*, quickness in "summing up"; *spatulate*, restlessness, mechanical power; *pointed*, loquacity, ruse, and ability to talk largely on small matters; *square*, reasonableness, sensibility displayed when necessary in speech and action. *Long in a good hand*, perseverance, intelligence, eloquence, philosophy; *very long*, scientific powers, the exercise of which will bring renown to the possessor.

